# Barriers and coping mechanisms to accessing healthcare during the COVID-19 lockdown: a cross-sectional survey among patients with chronic diseases in rural Rwanda

**DOI:** 10.1186/s12889-021-10783-z

**Published:** 2021-04-10

**Authors:** Alphonse Nshimyiryo, Dale A. Barnhart, Vincent K. Cubaka, Jean Marie Vianney Dusengimana, Symaque Dusabeyezu, Deogratias Ndagijimana, Grace Umutesi, Cyprien Shyirambere, Nadine Karema, Joel M. Mubiligi, Fredrick Kateera

**Affiliations:** 1 Partners In Health/Inshuti Mu Buzima (PIH/IMB), PO BOX 3432, Kigali, Rwanda; 2 Harvard Medical School, Boston, USA

**Keywords:** COVID-19 lockdown, Chronic care, Chronic diseases, Barriers and coping mechanisms to accessing healthcare, Rwanda

## Abstract

**Background:**

Large scale physical distancing measures and movement restrictions imposed to contain COVID-19, often referred to as ‘*lockdowns*’, abruptly and ubiquitously restricted access to routine healthcare services. This study describes reported barriers and coping mechanisms to accessing healthcare among chronic care patients during the nationwide COVID-19 lockdown in Rwanda.

**Methods:**

This cross-sectional study was conducted among chronic care patients enrolled in pediatric development, HIV/AIDS, non-communicable diseases, mental health, and oncology programs at 3 rural Rwandan districts. Active patients with an appointment scheduled between March–June 2020 and a phone number recorded in the electronic medical record system were eligible. Data were collected by telephone interviews between 23rd April and 11th May 2020, with proxy reporting by caregivers for children and critically ill-patients. Fisher’s exact tests were used to measure associations. Logistic regression analysis was also used to assess factors associated with reporting at least one barrier to accessing healthcare during the lockdown.

**Results:**

Of 220 patient respondents, 44% reported at least one barrier to accessing healthcare. Barriers included lack of access to emergency care (*n* = 50; 22.7%), lack of access to medication (*n* = 44; 20.0%) and skipping clinical appointments (*n* = 37; 16.8%). Experiencing barriers was associated with the clinical program (*p* < 0.001), with oncology patients being highly affected (64.5%), and with increasing distance from home to the health facility (*p* = 0.031). In the adjusted logistic regression model, reporting at least one barrier to accessing healthcare was associated with the patient's clinical program and district of residence. Forty (18.2%) patients identified positive coping mechanisms to ensure continuation of care, such as walking long distances during suspension of public transport (*n* = 21; 9.6%), contacting clinicians via telephone for guidance or rescheduling appointments (*n* = 15; 6.8%), and delegating someone else for medication pick-up (*n* = 6; 2.7%). Of 124 patients who reported no barriers to accessing healthcare, 9% used positive coping mechanisms.

**Conclusion:**

A large proportion of chronic care patients experienced barriers to accessing healthcare during the COVID-19 lockdown. However, many patients also independently identified positive coping mechanisms to ensure continuation of care - strategies that could be formally adopted by healthcare systems in Rwanda and similar settings to mitigate effects of future lockdowns on patients.

## Background

The coronavirus disease (COVID-19) pandemic has infected millions of people while also causing millions of deaths globally [1]. Historic health crises including the 2009 influenza A (H1N1) pandemic, the 2013–2016 West Africa Ebola outbreaks, and other natural disasters and humanitarian crises have overwhelmed health systems while disrupting the provision and access to routine healthcare [2–4]. These disruptions have been associated with poor patient outcomes, including increased morbidity, drug resistance, and mortality [4, 5]. Generally, efforts to contain the spread of COVID-19 have included national and regional lockdowns – defined as large scale physical distancing measures and movement restrictions. Current models suggest that disruption of routine health services due to COVID-19 pandemic and measures to control it could result in increased deaths from many non-COVID causes [6, 7]. These models estimate at least additional 253,500 child deaths and 12,200 maternal deaths [6], and 36, 20 and 10% increase in deaths due to malaria, TB and HIV, respectively [6, 7].

Health system disruption during the COVID-19 pandemic has been expected to particularly impact access to healthcare among chronic care patients in low- and middle-income countries (LMICs) [7, 8]. However, as has been seen in previous crises, these disruptions can be either exacerbated or mitigated through individual patient and health system responses. For instance, during political and economic crises and flood disasters in Zimbabwe and Namibia, patients receiving ART for HIV reported skipping drug doses, sharing and/or selling drugs and changing regimens [4, 9]. In contrast, during the 2008 political unrest in Kenya, patients and providers were able to create stockpiles of medication to mitigate treatment discontinuation [10]. During the COVID-19 pandemic, healthcare workers have adopted a variety of strategies, including telemedicine [11, 12] and home delivery of medication [13], to promote continuation of care without exposing patients to the risk of infection.

In Rwanda, the first case of COVID-19 was reported on March 14, 2020, and the Rwandan government rapidly enforced policies to stop local transmission, including the implementation of a national lockdown between March 22nd and May 3rd, 2020 [14]. During this lockdown, hospitals, clinics, health centers, pharmacies and other essential services remained open. However, patients’ access to health-care might have been highly affected by the geographical distribution of health facilities, tight restrictions on movements of people across subnational boundaries, suspension of public transport, and patients’ fear of COVID-19 infection [15]. During a rapid assessment conducted by the World Health Organization (WHO), national health organizations in all member states, including Rwanda, reported remarkable macro-level disruptions in non-communicable diseases services and resources due to COVID-19 [16]. However, this assessment did not capture chronic care patients’ individual experiences and did not assess experiences among rural patients, who may be most vulnerable to disruptions of care**.** During the March–April 2020 nationwide lockdown, Partners In Health/Inshuti Mu Buzima (PIH/IMB), a non-governmental organization that provides health system strengthening support to three rural districts in Rwanda, conducted a cross-sectional telephone survey among patients with chronic conditions who were receiving care from PIH/IMB-supported health facilities. In this paper, we describe patients’ barriers to accessing healthcare and coping mechanisms used by patients or their caregivers to ensure continuation of care during the lockdown in rural Rwanda.

## Methods

### Study setting

PIH/IMB is a non-profit organization that has been collaborating with the Rwanda Ministry of Health (MOH) to strengthen government owned health facilities since 2005 and currently supports facilities in Kayonza, Kirehe and Burera districts of Rwanda. These health facilities serve approximately one million people, including 24,635 patients with chronic conditions enrolled in five chronic care programs [17].

### Study design and population

This cross-sectional study targeted patients enrolled in one of five chronic care programs: a) the HIV program; b) the non-communicable diseases (NCD) program, which provides care for patients with type 1 and type 2 diabetes, asthma, hypertension and heart failure; c) the mental health (MH) program, which treats patients with depression and other mental health disorders as well as epilepsy; d) the pediatric development clinics (PDC), which provide clinical and nutritional follow-up to vulnerable children under-5 with developmental delays associated with various conditions, including prematurity, low birth weight and hypoxic ischemic encephalopathy [18]; and e) the oncology program. The HIV, NCD, and MH programs are currently active in all three districts; the PDC program is active in Kirehe and Kayonza districts whilst the oncology program is located in Burera district. Across all programs, study inclusion criteria were: (1) having a clinical appointment scheduled between March–June 2020, (2) having a contact telephone number recorded in the electronic medical record (EMR) system, and (3) being recorded in EMR as a resident in one of the 3 districts (Kayonza, Kirehe and Burera). However, as the proportion of HIV patients with a phone number recorded in EMR was particularly low, so we contacted 39 HIV patients through their community health workers (CHWs).

### Study sample and data collection

Data analyzed here were extracted from a survey conducted between April 23rd and May 11, 2020 among chronic care patients to inform provision of social support to these patients during the COVID-19 pandemic. To ensure sufficient sample sizes to investigate patient responses by clinical program and district, the survey used stratified random sampling. Twelve strata were defined by district and clinical program. The sample size estimate to allow to report 95% confidence intervals for program-specific estimates with a precision of +/− 15% was at least 48 patients from each clinical program (16 patients across 3 districts for HIV, NCD, and MH program, 24 patients across 2 districts for PDC, and 48 patients in one district for oncology). Initially, 75 patients from each clinical program were sampled for inclusion on first-call lists. If we were unable to attain our target stratum-specific sample size after contacting everyone on the first-call list, we sampled replacement patients from the same district-clinical program strata until we either reached our target sample size or all eligible individuals from that stratum had been contacted. Six data collectors with a strong research experience and familiarity with the chronic care programs collected the data. Computer-assisted telephone interviews were conducted, where the interviewer administered the survey questions by calling patients or their caregivers, while reading and directly recording responses in an electronic online form developed in REDCap electronic data capture tools hosted at PIH/IMB [19, 20]. Proxy reporting by caregivers was allowed for children and critically ill-patients. Data collectors were instructed to call every sampled patient at different times on 3 separate days before deciding that the patient could not be reached.

### Variables and data analysis

This study used data generated by the survey exploring effects of the COVID-19 lockdown on access to healthcare, patients’ psychological needs and patients’ or caregiver’s knowledge about the COVID-19 infection and its prevention as well as patients’ demographic and socio-economic characteristics. However, our analysis only focused on describing reported barriers and used strategies to accessing healthcare during the lockdown. Access to care questions were not validated scales, but instead reflected clinician and program managers’ perception about what barriers could be. Patients were invited to free-list barriers to care and strategies used to ensure continuation of care during the lockdown, but data collectors checked off pre-identified categories and provided patients with examples, as needed. “Other” was an option, but responses were evaluated and reclassified into one of the existing categories or created new categories. Socioeconomic status was measured using the 2015 Rwandan Government’s Ubudehe categorization of households - a four-level categorization ranging from 1 (poorest) to 4 (richest) [21, 22]. Category 1 included people who belong to families that don’t own a house and always struggle to afford basic needs. Families that own or are able to rent a house, but have members rarely getting fulltime jobs fall under the Ubudehe category 2. Category 3 included households that have members with a full time job or farmers who can go beyond subsistence farming. Families that own large-scale businesses or with individuals working with international organizations, industries, or as public servants are in Ubudehe category 4. Patients were asked to list all the effects that the lockdown had on their access to healthcare. *“Reporting at least one barrier to accessing healthcare”* was defined as the patient reporting one or more of the following effects of the lockdown: (i) lack of access to emergency care, (ii) skipping appointments, (iii) lack of access to medication, and (iv) difficulty getting (walking long distances due to suspension of public transport) to the health facility. Among patients who were prescribed at least one medication, interviewees were asked about their experiences in the 4 weeks prior to the survey that might have affected their ability to take their medication. *“Reduced ability to take medication as prescribed”* was explained by any of these reported experiences: a) feeling sad or depressed, b) running out of medication, c) unwillingness to take medication in the sight of other family members, and d) forgetting. Patients were also asked to name strategies they used to access health care during the lockdown. We classified these strategies as adverse coping mechanisms if they would prevent the continuity of care. *“Exclusive use of positive coping mechanisms”* was defined as the patient only reporting coping mechanisms that helped to maintain continuity of care. We used frequencies and percentages to describe patients and survey respondents’ characteristics, reported barriers, and coping mechanisms to accessing healthcare. In bivariate analyses, Fisher’s exact tests were used to measure associations between patient’s characteristics and reporting barriers to accessing healthcare. In addition, we conducted a multivariable logistic regression analysis to assess factors associated with reporting at least one barrier to accessing healthcare during the COVID-19 lockdown using Wald tests to calculate *p*-values for each variable in the logistic regression model. The association between utilized coping mechanisms to ensure continuation of care and reporting barriers to accessing healthcare during the lockdown was also assessed using Fisher’s exact test. The data were analyzed using Stata v.15.1 (Stata Corp, College Station, TX, USA).

## Results

**Fig. 1:**
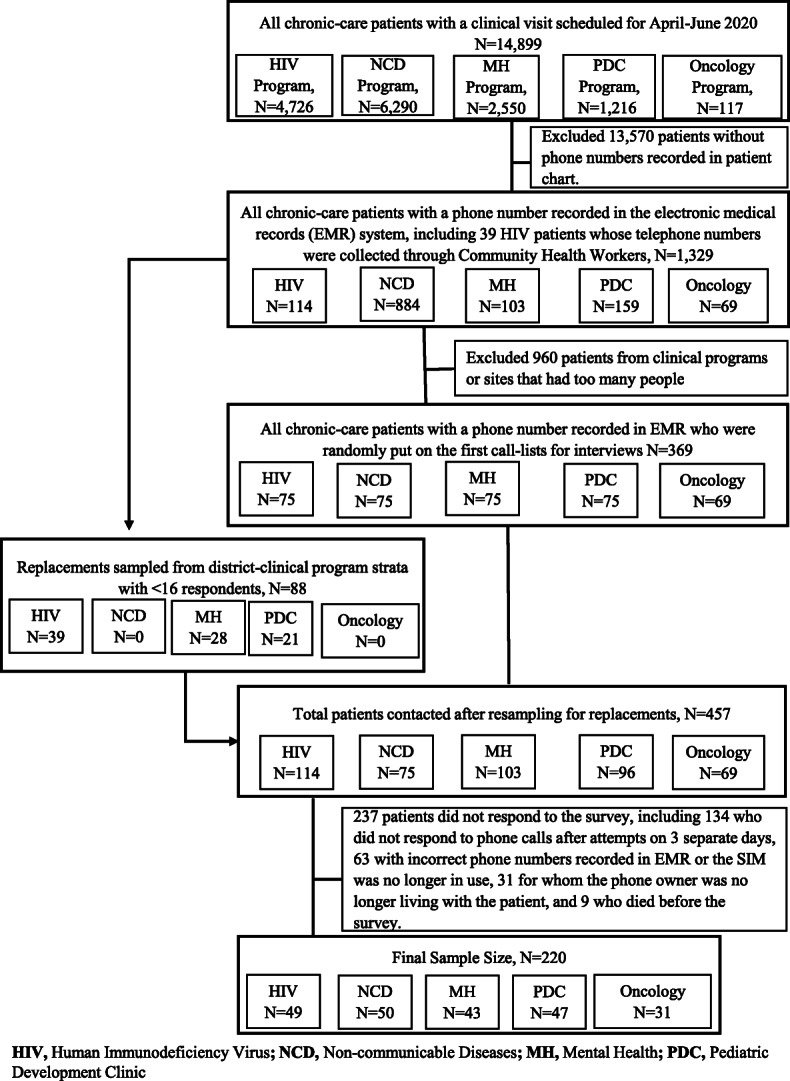
Flow chart of study participants

A total of 457 patients were identified from the electronic medical records (EMR) and contacted by telephone, including 369 patients from the first call-lists for interviews and 88 replacements resampled when the target sample size for the clinical program-district stratum had not been met (Fig. 1). Of these 457 patients, 220 (48.1%) responded. Response rates by clinical program were 43% for HIV, 67% for NCD, 42% for MH, 49% for PDC, and 45% for oncology. Non-response was due to either the patient not answering phone calls on 3 different attempts on 3 separate days (56.5%), to having incorrect phone numbers recorded in EMR (26.6%), the phone owner no longer living with the patient (13.1%) or the patient dying before the survey (3.8%).

Of the 220 patients who responded to the survey, 50 (22.7%) were from the NCD program, 49 (22.3%) from HIV, 47 (21.4%) from PDC, 43 (19.6%) were from MH, and 31 (14.1%) from oncology (Table 1). Sixty-eight percent of patients responded to the survey on their own behalf. Of 70 patients who had a caregiver respond on their behalf, 47 (67.1%) were children in PDC, 20 were (28.6%) MH patients, 2 (2.9%) were oncology patients and 1 (1.4%) was an HIV patient. Of 218 patients with known socioeconomic status, 38 (17.4%) were in the poorest category 1, 82 (37.6%) in category 2 and 98 (45.0%) in category 3. The estimated time from home to the facility of usual care was < 1 h for 78 (35.4%) patients and > 2 h for 42 (19.1%) patients. The majority of patients (77.7%) were prescribed medication to take at home.

**Table 1:** Respondents’ socio-demographic and patients’ clinical characteristics (*N* = 220)

Characteristic	N	%
Survey respondent		
Self	150	68.2
Caregiver	70	31.8
Patient/respondent’s level of education		
None	48	21.8
Primary	123	55.9
Secondary or higher	44	20.0
Missing data	5	2.3
Patient’s district		
Kayonza	75	34.1
Kirehe	80	36.4
Burera	65	29.6
Patient’s gender		
Male	83	37.7
Female	137	62.3
Patient’s age (years)		
< 5	28	12.7
5–17	9	4.1
18–35	34	15.5
36–59	79	35.9
> =60	36	16.4
Missing data	34	15.5
Patient’s marital status		
Married	81	36.8
Cohabiting	16	7.3
Widowed	28	12.7
Divorced	6	2.7
Single - adult	33	15.0
Child	56	25.5
Socio-economic status (Ubudehe category)		
1	38	17.3
2	82	37.3
3	98	44.6
Unknown	2	0.9
Patient’s clinical program		
HIV/AIDS	49	22.3
Non-communicable Disease (NCD)	50	22.7
Mental Health (MH)	43	19.6
Pediatric Development Clinic (PDC)	47	21.4
Oncology	31	14.1
Patient’s diagnoses^a^		
HIV/AIDS	50	22.7
Diabetes type 1	8	3.6
Diabetes type 2	9	4.1
Hypertension	47	21.4
Heart failure	2	0.9
Asthma	14	6.4
Cancer	32	14.6
Mental illnesses	43	19.6
PDC (Prematurity, low birth weight, hypoxic ischemic encephalopathy (HIE), etc.)	47	21.4
Was the patient prescribed medication to help manage health at home?		
No	49	22.3
Yes	171	77.7
Estimated time (in hours) from the patient’s home to the health facility of usual health-care		
< 1 h	78	35.4
1–2 h	100	45.5
> 2 h	42	19.1
Was the patient living with someone who could help remind the patient to take medication or accompany the patient to the health facility?		
No	49	22.3
Yes	167	75.9
Missing data	4	1.8

^a^It was possible for one patient to have multiple diagnoses

Forty-four percent of the patients reported at least one barrier to accessing healthcare during lockdown (Table 2). Reported barriers included lack of access to emergency care (*n* = 50, 22.7%), lack of access to medication (*n* = 44, 20.0%), skipping clinical appointments (*n* = 36, 16.4%) and lack of transport (*n* = 29, 13.2%). In the bivariate analyses, reporting barriers to accessing healthcare was associated with the clinical program (*p* = 0.001), with HIV/AIDS patients being least likely to report barriers to accessing healthcare (20.4%) and oncology patients being most likely to report barriers to accessing healthcare (64.5%). Reporting barriers to accessing healthcare was also associated with increased distance from home to the health facility of usual care (*p* = 0.031). In the adjusted logistic regression model, reporting at least one barrier to accessing healthcare was associated with the patient’s clinical program and district of residence. Higher odds of reporting at least one barrier to accessing healthcare were observed among patients in the oncology program [odds ratio (OR): 6.5; 95% confidence interval (CI): 1.9–21.8], NCD (OR: 3.7; 95% CI: 1.4–9.6), MH (OR: 3.6; 95% CI: 1.3–9.9) and PDC (OR: 3.4; 95% CI: 1.2–9.1) compared to HIV program patients. In addition, patients in Kirehe district had 2.1 times odds (95% CI: 1.0–4.4) of reporting at least one barrier to accessing healthcare compared to patients in Kayonza.

**Table 2:** Self-reported barriers to accessing healthcare for chronic-care patients during the COVID-19 lockdown in Rwanda (*N* = 220)

Variables	Bivariate analysis of the factors associated with reporting barriers to accessing healthcare during the COVID-19 lockdown					Multivariable logistic regression analysis of the factors associated with reporting at least one barrier to accessing healthcare in lockdown	
	Lack of access to emergency care, n (%)	Lack of access to medication, n (%)	Skipping clinical appointments, n (%)	Difficulty getting to the health facility (walking long distances, lack of transport), n (%)	At least one barrier to accessing health care reported, n (%)	Odds ratio [95% confidence interval]	***p***-value
Overall	50 (22.7)	44 (20.0)	37 (16.8)	28 (12.7)	96 (43.6)	–	–
Clinical program	(*p* = 0.239)	(*p* = 0.008)	(*p* < 0.001)	(*p* < 0.001)	(*p* = 0.001)		(*p* = 0.023)
HIV/AIDS	6 (12.2)	6 (12.2)	2 (4.1)	0 (0.0)	10 (20.4)	ref	
Non-communicable diseases	12 (24.0)	13 (26.0)	3 (6.0)	10 (20.0)	24 (48.0)	3.7 [1.4–9.6]	
Mental health	9 (20.9)	13 (30.2)	9 (20.9)	0 (0.0)	19 (44.2)	3.6 [1.3–9.9]	
Pediatric Development Clinic	14 (29.8)	3 (6.4)	15 (31.9)	6 (12.8)	23 (48.9)	3.4 [1.2–9.1]	
Oncology	9 (29.0)	9 (29.0)	8 (25.8)	11 (35.5)	20 (64.5)	6.5 [1.9–21.8]	
Estimated time (hours) from home to the health facility of usual health care	(*p* = 0.077)	(*p* = 0.465)	(*p* = 0.223)	(*p* = 0.006)	(*p* = 0.031)		(*p* = 0.243)
< 1 h	12 (15.4)	13 (16.7)	9 (11.5)	6 (7.7)	31 (39.7)	ref	
1–2 h	24 (24.0)	20 (20.0)	18 (18.0)	10 (10.0)	39 (39.0)	0.9 [0.4–1.7]	
> 2 h	14 (33.3)	11 (26.2)	10 (23.8)	12 (28.6)	26 (61.9)	1.9 [0.7–4.7]	
District of residence	(*p* < 0.001)	(*p* = 0.001)	(*p* = 0.104)	(*p* < 0.001)	(*p* = 0.073)		(*p* = 0.040)
Kayonza	12 (16.0)	7 (9.3)	11 (14.7)	16 (21.3)	29 (38.7)	ref	
Kirehe	33 (41.3)	26 (32.5)	19 (23.8)	1 (1.3)	43 (53.8)	2.1 [1.0–4.4]	
Burera	5 (7.7)	11 (16.9)	7 (10.8)	11 (16.9)	24 (36.9)	0.8 [0.4–1.9]	
Socio-economic status (Ubudehe category)^a^	(*p* = 0.535)	(*p* = 0.690)	(*p* = 0.506)	(*p* = 0.058)	(*p* = 0.121)		(*p* = 0.068)
1	6 (15.8)	7 (18.4)	8 (21.1)	3 (7.9)	13 (34.2)	ref	
2	21 (25.6)	14 (17.1)	11 (13.4)	8 (9.8)	32 (39.0)	1.3 [0.5–3.0]	
3	22 (22.5)	22 (22.5)	18 (18.4)	17 (17.4)	50 (51.0)	2.4 [1.0–5.9]	
Living with someone who could help remind the patient to take medication or accompany the patient to the health facility^b^	(*p* = 0.437)	(*p* = 0.416)	(*p* > 0.999)	(*p* = 0.469)	(*p* = 0.624)		(*p* = 0.843)
No	13 (26.5)	12 (24.5)	8 (16.3)	8 (16.3)	23 (46.9)	ref	
Yes	35 (21.0)	31 (18.6)	27 (16.2)	20 (12.0)	71 (42.5)	1.1 [0.5–2.3]	

^a^Two patients didn’t have data on socio-economic status

^b^Four patients had missing data on whether they were living with someone who could remind the patient to take medication/accompany the patient to the health facility or not

Of 171 patients who were prescribed medication to take at home during the lockdown, 89 (52.1%) reported reduced ability of taking that medication as prescribed (Table 3). The most common reasons for not adhering to their medication prescription included self-reported feeling sad or depressed (*n* = 61, 35.7%), running out of medication (*n* = 42, 24.6%) and being unwilling to take medication in the sight of other family or household members (*n* = 27, 15.8%). Reporting reduced ability to take medication as prescribed during the lockdown was significantly associated with the patient’s district of residence (*p* < 0.001), with patients living in Kirehe district being highly affected (*n* = 45, 77.6%). Living with someone who could help remind the patient to take medication or accompany the patient to the health facility did not make a significant difference with regard to the patient’s ability to take medication as prescribed.

**Table 3:** Self-reported factors negatively affecting the patient’s ability of taking medication at home as prescribed during COVID-19 lockdown (*N* = 171)

Variables	Reduced ability to take medication at home as prescribed, n (%)	Feeling sad or depressed, n (%)	Ran out of medication, n (%)	Unwilling to take medication in the sight of other family/household members, n (%)	Forgetting, n (%)
Overall	89 (52.1)	61 (35.7)	42 (24.6)	27 (15.8)	22 (12.9)
Clinical program	(*p* = 0.920)	(*p* = 0.487)	(*p* = 0.022)	(*p* = 0.080)	(*p* = 0.232)
HIV/AIDS	25 (51.0)	18 (36.7)	6 (12.2)	14 (28.6)	9 (18.4)
Non-communicable diseases	26 (54.2)	20 (41.7)	11 (22.9)	4 (8.3)	3 (6.3)
Mental health	22 (51.2)	15 (34.9)	13 (30.2)	5 (11.6)	7 (16.3)
Pediatric Development Clinic	3 (75.0)	2 (50.0)	3 (75.0)	0 (0.0)	1 (25.0)
Oncology	13 (48.2)	6 (22.2)	9 (33.3)	4 (14.8)	2 (7.4)
Estimated time (hours) from home to the health facility of usual health care	(*p* = 0.317)	(*p* = 0.482)	(*p* = 0.557)	(*p* = 0.891)	(*p* = 0.549)
< 1 h	24 (43.6)	16 (29.1)	13 (23.6)	8 (14.6)	6 (10.9)
1–2 h	45 (55.6)	31 (38.3)	18 (22.2)	14 (17.3)	13 (16.1)
> 2 h	20 (57.1)	14 (40.0)	11 (31.4)	5 (14.3)	3 (8.6)
District of residence	(*p* < 0.001)	(*p* < 0.001)	(*p* < 0.001)	(*p* < 0.001)	(*p* = 0.002)
Kayonza	15 (27.8)	6 (11.1)	7 (13.0)	1 (1.9)	3 (5.6)
Kirehe	45 (77.6)	35 (60.3)	26 (44.8)	18 (31.0)	15 (25.9)
Burera	29 (49.2)	20 (33.9)	9 (15.3)	8 (13.6)	4 (6.8)
Socio-economic status (Ubudehe category)^a^	(*p* = 0.863)	(*p* = 0.892)	(*p* = 0.613)	(*p* = 0.485)	(*p* = 0.324)
1	17 (53.1)	12 (37.5)	7 (21.9)	3 (9.4)	3 (9.4)
2	34 (54.0)	23 (36.5)	13 (20.6)	12 (19.1)	11 (17.5)
3	37 (49.3)	25 (33.3)	21 (28.0)	11 (14.7)	7 (9.3)
Living with someone who could help remind the patient to take medication or accompany the patient to the health facility^b^	(*p* = 0.598)	(*p* > 0.999)	(*p* = 0.288)	(*p* = 0.336)	(*p* = 0.440)
No	24 (55.8)	15 (34.9)	12 (27.9)	9 (20.9)	7 (16.3)
Yes	64 (50.4)	46 (36.2)	29 (22.8)	18 (14.2)	15 (11.8)

^a^Two patients didn’t have data on socio-economic status

^b^Four patients had missing data on whether they were living with someone who could remind the patient to take medication/accompany the patient to the health facility or not

A total of 77 (35%) patients reported using either adverse or positive coping mechanisms in response to the effects of the lockdown (Table 4). Patients who reported at least one barrier to health care were more likely to adopt negative coping mechanisms, such as skipping or delaying appointments than those who did not (24.0% vs. 11.3%). Eighteen percent of patients used exclusively positive coping mechanisms to ensure continuation of care during the lockdown. These positive coping mechanisms included walking long distances due to suspension of public transport (*n* = 21, 9.6%), contacting the usual clinician via telephone for guidance or rescheduling the appointment (*n* = 15, 6.8%), delegating relatives or neighbor clinicians to pick up medication for the patient (*n* = 6, 2.7%), going to a community health worker (CHW) (n = 2, 0.9%), and buying medication from a nearest pharmacy (n = 1, 0.5%). Of 124 patients who reported no barriers to accessing healthcare, 11 (8.9%) reported using any of the positive coping mechanisms. Patients reporting barriers to accessing healthcare were still more likely to use positive coping mechanisms than those who did not. There was no association between the patient’s socio-economic status and using positive/negative coping strategies (results not presented in tables).

**Table 4:** Self-reported coping mechanisms and association with reported barriers to accessing health-care during the COVID-19 lockdown

Reported coping mechanisms	Overall, ***N*** = 220	Among patients reporting no barrier to accessing health care, ***N*** = 124	Among patients reporting at least 1 barrier to accessing health care, ***N*** = 96	***p***-value
	n (%)	n (%)	n (%)	
**Adverse coping mechanisms:**				
Stopped treatment, skipped, or delayed treatment	37 (16.8)	14 (11.3)	23 (24.0)	0.018
**Positive coping mechanisms**				
Went on foot or used other alternative forms of transport to go to the health facility	21 (9.6)	7 (5.7)	14 (14.6)	0.036
Contacted usual clinician via phone for guidance/to reschedule appointment	15 (6.8)	2 (1.6)	13 (13.5)	0.001
Delegated son/daughter/neighbour clinician to pick up medication for me	6 (2.7)	0 (0.0)	6 (6.3)	0.006
Went to a community health worker (CHW)	2 (0.9)	2 (1.6)	0 (0.0)	0.506
Bought medication from a nearest pharmacy	1 (0.5)	0 (0.0)	1 (1.0)	0.436
**Used exclusively positive coping mechanisms**	40 (18.2)	11 (8.9)	29 (30.2)	< 0.001

## Discussion

In this study, we described barriers and coping mechanisms to accessing healthcare among rural patients with chronic diseases who required chronic care during a nationwide COVID-19 lockdown in Rwanda. A large proportion (44%) of patients reported barriers to accessing healthcare, while about 18% of patients were able to identify positive coping mechanisms that helped to ensure continuation of care during the lockdown. Despite the adoption of positive coping mechanisms, patients who reported barriers to healthcare access were still more likely to skip appointments or delay treatment than those who did not. Avoiding barriers to healthcare access during lockdowns remains critical for chronic care patients since non-adherence to medical treatment is particularly associated with worse treatment outcomes for these patients [23].

Although health facilities in Rwanda remained open for both emergency and routine services throughout the duration of the lockdown, 22.7% of patients still reported being unable to access emergency care and 16.8% reported being unable to attend regular clinical appointments. These results indicate a reduced access to emergency care and attendance of scheduled medical appointments due to the COVID-19 lockdown that is consistent with evidence from other settings [24–29]. Perceived reasons for a dramatic decline in the utilization of emergency care services and low attendance of medical visits during the COVID-19 pandemic varied by setting, and included absence of public transport or increased costs of transport [24], shortage of care providers due to reallocation of staff to the COVID-19 response [25], and fear of COVID-19 infection [26–28].

This study also revealed a significant association between experiencing barriers to accessing healthcare and the patient’s clinical program. The lowest proportion of patients reporting barriers to accessing healthcare was observed among patients in the HIV program, while patients in all other chronic care programs had higher odds of reporting barriers to accessing healthcare during the COVID-19 lockdown compared to HIV program patients. None of the HIV patients reported lack of transport as an issue and HIV patients were also the least likely to report running out of medication as a barrier to taking medications as prescribed. Unlike PDC, Oncology, MH and NCD clinics, HIV treatment program has been decentralized to the health center level in all PIH/IMB-supported districts, which is typically within walking distance for patients [30]. Additionally, in 2018 PIH/IMB-supported districts implemented the HIV differentiated service delivery model (DSDM) launched by the Rwanda Ministry of Health. This strategy entails having stable HIV patients attend one clinical visit per 6 months and receive ARVs and OIs prophylaxis medications for a 3-month period. The low effect of the COVID-19 lockdown on HIV program patients also suggests the importance of DSDM that aimed at more frequent services for higher-risk HIV patients and less frequent routine appointments for stable, low-risk HIV patients by offering them drug refill every 3 months and appointments every 6 months.

In contrast, access to oncology was badly affected by the lockdown. Until the onset of COVID-19, oncology services in PIH/IMB-supported districts were still centralized and only available at Butaro District Hospital. Therefore, patients in the oncology program traveled longer distances to access care compared with patients in other programs. Suspension of public transport during the lockdown would have also made it difficult for oncology patients to access emergency care and attend scheduled clinical appointments. In general, we observed that increasing distance to the health facility was also associated with experiencing barriers to accessing healthcare. These findings suggest that decentralized treatment programs may be less vulnerable to disruptions due to COVID-19 and join a larger body of research highlighting the importance of treatment decentralization in promoting patient’s access to health care [31, 32].

In contrast to a previous study in Nigeria, which reported that 35% of patients with chronic diseases had difficulties accessing medication during the COVID-19 lockdown [33], we found that a relatively low rate (20.0%) of patients with chronic conditions reported lack of access to medication due to the COVID-19 lockdown [33, 34]. However, 50.3% of patients who were prescribed medication still reported reduced ability to take their medication at home as prescribed. The most common reason for reduced ability of taking medication at home was feeling sad or depressed. It has been expected that the COVID-19 pandemic could increase the levels of anxiety and depression [35], and there is a strong evidence that depression among chronic care patients negatively affects adherence to medical treatment [36, 37]. During the pandemic, PIH/IMB and other care providers may need to provide additional socio-economic and psychological support to improve the mental health of patients and therefore increase medication adherence.

Many patients facing barriers to accessing healthcare independently identified coping mechanisms to ensure continuation of care during the lockdown. Two of these strategies, contacting clinicians via telephone and delegating relatives or neighbors to pick up medication for the patient, could be formalized to promote continuity of care during future crises. Although telephone consultations cannot replace clinical consultations, given the increase in access to mobile phones in low-income countries, health systems could leverage the use of these tools to accelerate the provision and access to care through telemedicine [11, 12, 38]. In our setting, as in other rural areas in Rwanda, access to mobile phones is still relatively low (58.6% in rural vs. 87.9% in urban) [39] and patients’ contact information change often, so physician-initiated phone calls is not currently feasible. However, establishing a clinical program helpline for patients and sharing this number with patients and their community health workers, who are provided with phones, could formalize this line of communication. Additionally, programming the EMR to provide annual reminders to update patients’ telephone numbers may improve the feasibility of future clinician-initiated communication. Similarly, allowing patients to designate formal delegates to obtain medication refills could be especially helpful for patients on multi-month prescriptions, who must still visit the pharmacy for monthly refills due to medical insurance requirements.

Although many patients reported walking long distances due to suspension of public transport, this positive coping mechanism is not a solution that should be formalized, since this strategy would not be accessible to all patients, especially the elderly or disabled. Health providers may instead work with local authorities to provide transport passes for patients’ appointments or increase decentralization of services.

This study had several limitations. First, our survey respondents’ population may not be representative of our patient population. Due to safety considerations and travel restrictions during the COVID-19 lockdown, our study population was limited to patients with active phone numbers, and only 9% of eligible patients had a phone number recorded in EMR. We hypothesize that this selection bias may have resulted in a respondent population that was wealthier than the overall patient population served by PIH/IMB. However, the magnitude of this bias may not be large because the proportion of survey respondents in the lowest Ubudehe category 1 is very similar to rates reported in the general population in Rwanda (17.3% vs. 16.0%) [40]. Second, this study only included patients who were residents in three districts supported by PIH/IMB and the effects of COVID-19 on patients in PIH/IMB-supported areas may differ from effects in other areas of Rwanda. These biases would likely result in an underestimate of the negative effects of the lockdown on chronic care patients, and our results should be viewed as a lower bound of the likely negative effects of the COVID-19 pandemic on rural Rwandan populations. These results may also not be generalizable to patients living in urban areas, who would have been living in areas with higher COVID-19 transmission but would have also had easier access to a wider variety of public and private health facilities within walking distance as well as better access to private transport options.

Our findings reveal that during the nationwide COVID-19 lockdown, a significant proportion of chronic care patients in rural Rwanda experienced barriers to accessing healthcare and challenges adhering to medication. In general, our data suggests the impact of the lockdown were smallest for patients from highly decentralized programs, like HIV, and worst for highly centralized programs that required patients to travel long distance, like oncology. To promote resilience and help mitigate effects of future lockdowns or other crises, we recommend healthcare systems in Rwanda or other similar settings consider a) continue to pursue decentralization to enhance access to health services b) leverage mobile phones as a formal mean of communication between patients and clinicians, and c) allow patients to designate delegates to obtain medication refills. We also recommend further research to explore the patients’ experience and motivation to engage in the behavior that helped to cope with the barriers to accessing healthcare during the COVID-19 lockdown.

## Data Availability

The datasets used and/or analysed during the current study are available from the corresponding author on reasonable request.
